# The College of Nursing in Scotland

**Published:** 1918-07-13

**Authors:** 


### THE COLLEGE OF NURSING IN SCOTLAND.

Interest in the College of Nursing grows steadily in Scotland. Dr. D. J. Mackintosh, C.B., medical superintendent of the Western Infirmary, Glasgow, who has been chairman of the Registration Committee of the Scottish Board of the College from the commencement, informs us that the number of applicants for membership of the College is steadily increasing there. In view of the discussion and the Speaker's comment on Dr. Chapple's action in the House of Commons in regard to the London Hospital's training, Dr. Mackintosh claims that Scotland secured the insertion of the word "general" in Clause 10 of the Nurses' Registration Bill, seventh draft, approved by the Council on the 20th ult., which now reads, "and has had not less than three years' general training under a definite curriculum in a nurse-training school or schools recognised by the General Nursing Council and has passed its prescribed examinations." Lord Knutsford's letter on the proceedings in the House of Commons emphasises the desirability and importance for every nurse-training school to bring its system into line with Clause 10 just given.

